# Bilateral Bronchiectasis as a Presentation Form of Pulmonary Marginal Zone B-Cell Lymphoma of Bronchus Associated Lymphoid Tissue

**DOI:** 10.1155/2015/975786

**Published:** 2015-12-29

**Authors:** Glenda Ernst, Carla Torres, Eduardo Borsini, Félix Vigovich, Daniel Downey, Alajandro Salvado, Martín Bosio

**Affiliations:** ^1^Hospital Británico de Buenos Aires, C1280AEB Buenos Aires, Argentina; ^2^Hospital San Juan de Dios, Buenos Aires, Argentina; ^3^Hospital Español, Buenos Aires, Argentina

## Abstract

The pulmonary marginal zone B-cell lymphoma of bronchus associated lymphoid tissue of the lung (BALT) is a rare illness that can remain without symptoms. Radiological findings of pulmonary lymphoma are heterogeneous. In literature, bronchiectasis is only described in one patient who also had besides adenomegalies. We reported on a 48-year-old female patient. She showed symptoms consistent with dyspnea with productive cough; there were crepitant sounds in the auscultation. Pulmonary functional test has shown a severe restrictive pattern with a low FVC and DLCO. CT scan showed bronchiectasis in the medium lobule without adenomegalies. Echocardiogram was normal, and the laboratory findings only showed leukocytosis. There were no findings in the bronchoscopy, but the lung biopsy showed a B-cell pulmonary lymphoma (positive to CD20 and CD79a in immunostaining). A wide variety of radiological manifestations has been previously described; however, we have presented this rare case, with bronchiectasis, as unique radiological finding.

## 1. Introduction

Pulmonary marginal zone B-cell lymphoma of bronchus associated lymphoid tissue of the lung (BALT) lymphoma is characterized by an abnormal infiltration of lymphoid cells in the lung parenchyma. These pulmonary primary neoplasms are commonly localized in the extranodal marginal zone tissue without extrathoracic involvement at the beginning of the illness [1]. BALT constitutes an infrequent entity representing only the 0.5% of pulmonary primary neoplasm; however, it is the major type of lung lymphoma (between 72 and 90%) [2].

Radiological manifestation of BALT lymphomas has included multiple pulmonary nodules, pulmonary tumors, and consolidative areas [3]. Although cavitation and pleural involvement are rare, these findings have been described in a reduced number of patients [4].

We present a particular patient, who showed bilateral bronchiectasis in the CT scan and was diagnosed with pulmonary BALT lymphoma.

## 2. Case Presentation

A 48-year-old female patient with BMI of 23.3 is without illnesses history, is nonalcoholic, and is not smoker or exposed to other toxic expositions. She went to consulting room with dyspnea (mMRC: 3) that had begun to affect her daily activities. She also presented with productive cough, which has lasted the last two months.

Despite this, the patient presented a good health state and was without fever at the time of the consulting. The auscultation in the pulmonary examination showed crepitated sounds, with a good oxygen saturation (95% on room air). No other evidences were found.

The laboratory findings showed leukocytosis. The acute face reactants were normal (VSG and PCR). AISD and Koch bacillus culture were negative. Immunological panel: FR, antibodies for DNA, FAN, C3, C4, C-ANCA, and P-ANCA were also negative. Values of *α*1-antitrypsin and *β*2-microglobulin were normal.

Pulmonary functional test had shown a severe restrictive pattern with a diminished vital capacity (FVC) and diffusion capacity (DLCO) (Table 1). A six-minute walking test was performed in which the patient walked 500 meters with an initial saturation of 93% (BORG 0) and finishing with 86% (BORG 3).

**Figure 1:**
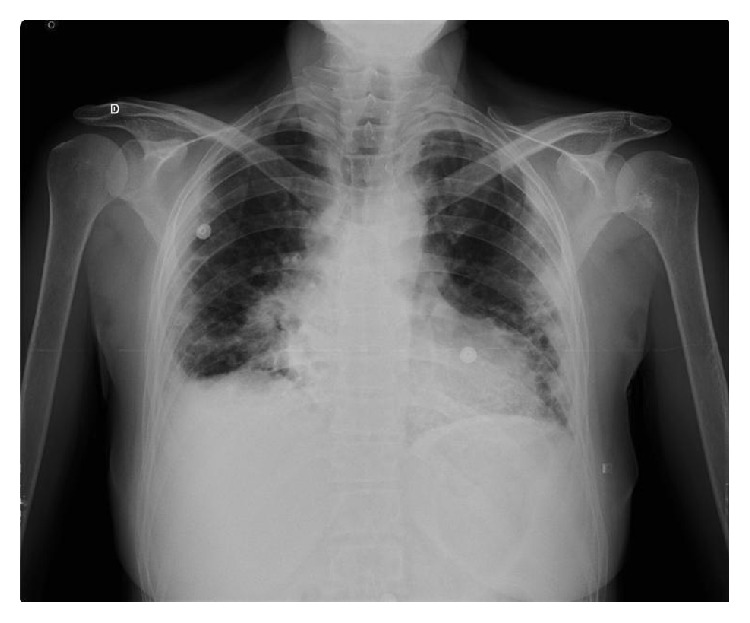
Loss of volume observed with peripheral infiltrates and pleural bilateral involvement.

**Figure 2:**
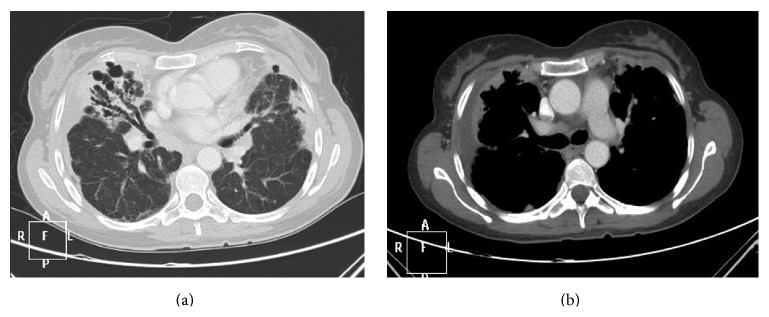
CT scan showing bronchiectasis in the medium and lower lobule and bilateral pleural thickening (a), without adenomegalies (b).

Chest X-ray revealed images with bilateral opacities with right pleural effusion (Figure 1). CT scan showed bronchiectasis in the medium and lower lobule and pleural bilateral thickening (Figure 2(a)), without adenomegalies (Figure 2(b)). PET-scan showed SUV of 10.4 with involvement of parenchyma in the medium lobule and supradiaphragmatic nodules.

Echocardiogram was normal, systolic functions in both ventricles (FSVI and FSVD) were conserved, TAPSE was 22 mm, and PSAP was 25 mmHg. The filled pattern was normal.

Fibrobronchoscopy was performed without evidencing abnormal findings in the trachea or bronchi. The cytology of bronchoalveolar lavage was negative for tumoral cells and cellular count was normal. The bacteriological cultures were also negative.

**Figure 3:**
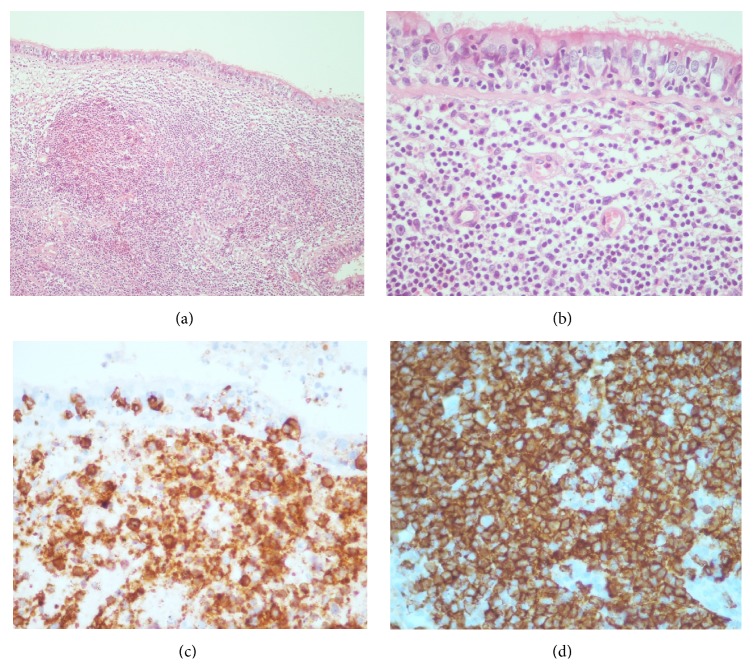
Lung biopsies. (a) Bronchial mucosa with lymphoid infiltration by small cells with germinal center formation. (40x-HE). (b) Lymphoepithelial lesions (100x-HE). Immune-histochemical demonstrating that lymphocytes were positive for BCL-2 (c) and CD20 (d).

Pulmonary and mediastinum lymph node biopsies were carried out. Histopathology findings revealed a lymphoid proliferative process in the lung parenchyma; the lymphocytes were immune-histochemically positive for CD20 and CD79a, PAX5, and BCL2 and negative for CD3, CD5, CD23, CD10, and cyclin-D1. These findings have contributed to demonstrate a B-cell lymphoma consistent with marginal zone lymphoma of the lung. Pulmonary and mediastinal lymph nodes showed follicular lymphoid hyperplasia without lymphomatous changes. Bone marrow biopsy did not show an involvement of the disease (Figure 3).

Patient started the treatment with rituximab for 12 months, showing an improvement of clinical symptoms. Currently (18 months after), she is being continuously examined as out-patient, showing PET-scans control with SUV 2.5.

## 3. Discussion

Primary lung lymphoma is a rare illness that presents different pathological variants and degrees of malignancy: most B-cell lymphomas are low grade malignancy and they are usually associated with the bronchial mucosa (BALT lymphomas/MALT) and represent about 90% of all primary lung lymphomas; large B-cell lymphomas show an intermediate to high grade malignancy; and finally peripheral T cell lymphomas occur as part of a systemic process with lymphoid granulomatosis or peripheral T cell lymphomas [3].

Most of the patients remain without symptoms until diagnosis (between 37.5 and 50%). Usually, the symptoms are nonspecific like cough, dyspnea, thoracic pain, and hemoptysis. Extrapulmonary manifestations are unusual but include fever, weight loss, and nocturnal sweating. Acute reactants as PCR, ERS, or LDH could be increased; however, these findings are nonspecific [5].

To diagnose pulmonary primary lymphoma criteria established by Saltzstein that show unilateral or bilateral pulmonary involvement should be kept, having mediastinal or hilar lymphadenopathy or not, and it must not have evidence of extrathoracic disease within 3 months of diagnosis [6].

The pathogenesis of this illness remains unclear; however, chronic antigenic stimulation, as in autoimmune disease, infection, and/or smoking, has been suggested as a trigger, which supports the fact that deregulation of lymphoid cell function may be involved [7, 8].

In 30–40% of patients autoimmune diseases (Sjögren's syndrome, rheumatoid arthritis, systemic lupus erythematosus, pernicious anemia, and Hashimoto thyroiditis), immunodeficiency states such as AIDS or the common variable immunodeficiency, and HCV infection could also present [9].

Radiological findings of pulmonary lymphoma are heterogeneous; these are characterized by consolidations with well-defined mass or reticulonodular pattern [3]. Computed tomography usually shows findings in the parenchyma or on the peripheral zone especially in the lower lobes. Between 60 and 70% of the cases show bilateral involvement, while between 70 and 77% of the cases have got single or multiple nodules with consolidated areas or cysts. The commonest presentation includes findings such as masses with well-defined margins or with air bronchogram. For this reason, the main proposed differential diagnosis would be the bronchoalveolar carcinoma [10, 11].

Only one patient with bronchiectasis and bilateral hilar and mediastinal lymph node enlargement has been previously described. However, the physicians initially suspected in the diagnosis of sarcoidosis [10]. According to this case, our patient showed bronchiectasis in the medium lobule, lingula, and pleural bilateral thickening but without adenomegalies to support another differential diagnosis.

Other associated features include angiogram sign and ground glass halo in thickening peribronchovascular area, especially in the margins of the tumor [12].

Hilar or mediastinal lymphadenopathies are less common and their presence would be suggesting dissemination of the disease and would be associated with bad prognosis. Nevertheless, pleural and vascular involvements have not been associated with poor prognosis. The cavitation of nodules is extremely rare [4, 13]. Bronchoalveolar lavage contributes to finding lymphoid infiltration in about 65% of cases [5], although the immunohistochemistry to diagnose such illness is necessary. These cells express B-cell markers, such as CD20 and CD79a, and are negative for CD5, CD10, CD23, and cyclin-D1 [14].

Therapeutic strategies include surgical resection followed by chemotherapy and/or radiotherapy. The choice will depend on the stage of the tumor; however, treatment in patients with limited disease remains not well established. It has been suggested that patient coursing the initial diagnosis of BALT without symptoms could be observed and followed up without treatment [15]. However, this indication is controversial [16]. The benefits of lung surgery and radiation therapy are questionable because some patients have presented with complications as thoracic pain and lung function impairment [17]. Until now, chemotherapy with chlorambucil would be the best treatment option for cases with disseminated disease [18]; however, another option is treatment with rituximab in patients with cells CD20+ [19].

To conclude, pulmonary BALT lymphoma is a rare tumor with a variety of radiological manifestations. The consolidation and multiple pulmonary nodules are the most frequent; however, we have presented this rare case, with bronchiectasis, as unique radiological finding.

## Figures and Tables

**Table 1:** Pulmonary function test.

	Pulmonary function test
	After bronchoscopy
FVC (L)	1.61
FVC (%)	42
FEV1 (L)	1.52
FEV1 (%)	50
FEV1/FVC (%)	95

TLC (L)	2.8
DLCO unc (mL/min/mmHg)	13.88
DLCO unc (%)	50
